# Regulation of MntH by a Dual Mn(II)- and Fe(II)-Dependent Transcriptional Repressor (DR2539) in *Deinococcus radiodurans*

**DOI:** 10.1371/journal.pone.0035057

**Published:** 2012-04-16

**Authors:** Hongxing Sun, Mingfeng Li, Guangzhi Xu, Huan Chen, Jiandong Jiao, Bing Tian, Liangyan Wang, Yuejin Hua

**Affiliations:** 1 Key Laboratory for Nuclear-Agricultural Sciences of Chinese Ministry of Agriculture and Zhejiang Province, Institute of Nuclear-Agricultural Sciences, Zhejiang University, Hangzhou, China; 2 Key Laboratory of Laboratory Medicine, Ministry of Education, Zhejiang Provincial Key Laboratory of Medical Genetics, School of Laboratory Medicine & Life Science, Wenzhou Medical College, Wenzhou, Zhejiang, China; 3 Zhejiang Institute of Microbiology, Zhejiang Province, Hangzhou, China; Louisiana State University and A & M College, United States of America

## Abstract

The high intracellular Mn/Fe ratio observed within the bacteria *Deinococcus radiodurans* may contribute to its remarkable resistance to environmental stresses. We isolated DR2539, a novel regulator of intracellular Mn/Fe homeostasis in *D. radiodurans*. Electrophoretic gel mobility shift assays (EMSAs) revealed that DR2539 binds specifically to the promoter of the manganese acquisition transporter (MntH) gene, and that DR0865, the only Fur homologue in *D. radiodurans*, cannot bind to the promoter of *mntH*, but it can bind to the promoter of another manganese acquisition transporter, MntABC. β-galactosidase expression analysis indicated that DR2539 acts as a manganese- and iron-dependent transcriptional repressor. Further sequence alignment analysis revealed that DR2539 has evolved some special characteristics. Site-directed mutagenesis suggested that His98 plays an important role in the activities of DR2539, and further protein-DNA binding activity assays showed that the activity of H98Y mutants decreased dramatically relative to wild type DR2539. Our study suggests that *D. radiodurans* has evolved a very efficient manganese regulation mechanism that involves its high intracellular Mn/Fe ratio and permits resistance to extreme conditions.

## Introduction

*Deinococcus radiodurans* belongs to the *Deinococcaceae* family of bacteria. These bacteria are characterized by extreme resistance to ionizing radiation (IR), ultraviolet (UV) radiation, oxidative stress, and desiccation [1]–[3]. To evaluate its robust viability in extreme environments, extensive studies on its ability to repair its DNA have being carried out [4], [5]. However, more and more evidence supports the view that proteins, especially those involved in DNA repair and replication, must be protected from damage by radiation toxicity before DNA repair can begin. The antioxidants present in *D. radiodurans* seem to play critical roles in cell survival during irradiation. Recent studies have demonstrated that Mn-dependent antioxidant mechanism can protect proteins from reactive oxygen species (ROS)-induced damage in many species, including *D. radiodurans* [6]–[8]. *D. radiodurans* contains approximately 100 times more Mn(II) than *Escherichia coli* when grown in a defined minimal medium. This suggests that the regulation of Mn(II) homeostasis in *D. radiodurans* may be more complicated than in *E. coli* [9].

So far, two types of bacterial high-affinity manganese acquisition systems have been identified, the natural resistance-associated macrophage protein (Nramp) transporter and the ATP-binding cassette (ABC) transporters [10], [11]. Both Nramp and ABC-type acquisition system homolog have been found in *D. radiodurans* [12], [13]. Nramp is a ubiquitous proton-coupled divalent metal ion transporter, and bacterial homologues have been reported in *Mycobacterium tuberculosis*, *Bacillus subtilis*, *Escherichia coli*, and *Salmonella enteric* serover Typhimurium [10], [14]–[16]. The primary role of bacterial Nramp homologues is to transport Mn(II), so it was renamed MntH (manganese transport, H-dependent) [10], [11], [14]. Manganese is an essential enzyme cofactor. It protects cells from oxidative damage. However, it can be toxic at high concentrations, and so the expression of MntH must be strictly regulated [6], [17]. The regulation of *mntH* genes in response to Mn(II) availability is mediated by the manganese transport regulator (MntR), among other regulators, including Fe(II)-sensing ferric uptake repressor (Fur) and the peroxide-sensing OxyR factor [10], [16], [18], [19]. MntR is a diphtheria toxin repressor (DtxR) homologue. It represses the transcription of genes encoding manganese acquisition systems, such as *mntH* and *mntABCD* in *B. subtilis*. Unlike other DtxR family members, which can sense both Mn(II) and Fe(II), the MntRs in *B. subtilis*, *E. coli*, and *S. aureus* exhibit pronounced selectivity for Mn(II) due to amino acid substitutions at the metal-binding site [20]–[23].

In *D. radiodurans*, *dr1709* encodes the manganese transporter, which is believed to be responsible for its high Mn(II) accumulation in *D. radiodurans* [7]–[9], [13]. Unfortunately, *dr1709* could not be disrupted and its repression is still unknown [12]. *dr2539* encodes a DtxR homologue, and previous studies have implied that it may regulate the intracellular Mn/Fe ratio [24]. However, which genes DR2539 regulates and the mechanism by which it regulates them remains to be investigated. In this study, we successfully expressed the DR2539 *in vivo* and analyzed its biochemical characteristics both *in vivo* and *in vitro*. We found that the transcription of *dr1709* was strictly repressed by DR2539. Unexpectedly, the Fe(II)-induced repression of *dr1709* was also found to be mediated by DR2539 but not by the Fur homologue DR0865. We demonstrated that DR2539 can be activated by both Fe(II) and Mn(II) *in vitro* and *in vivo*. Site-directed mutagenesis analysis showed that His98 plays an important role in the DNA binding activity of DR2539. Our study suggests that the metal binding mode of DR2539 may be different from what has been previously reported for the MntH repressor. It may represent a new subset of DtxR family proteins.

## Results

### Binding of DR2539 to the *mntH* (*dr1709*) promoter

**Figure 1:**
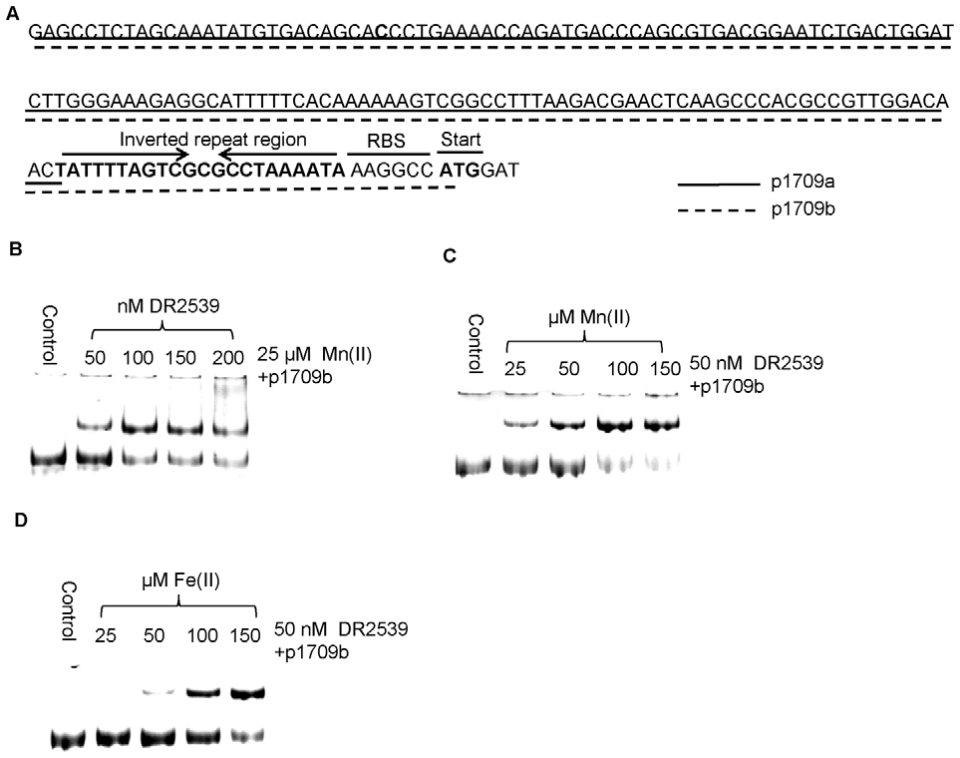
DR2539 binds to the MntH promoter DNA fragment in a Mn(II)- and Fe(II)-dependent manner. (A) Schematic of *dr1709* promoter (p1709a and p1709b) DNA sequence region. The inverted repeat region is shown by the inverted arrows. (B) DR2539 binding to p1709b with increasing quantities of DR2539 and 25 µM Mn(II). (C) and (D) EMSA analysis was performed using DR2539 and p1709b with increasing concentration of Mn(II) or Fe(II). RBS, ribosome binding site; Start, transcription start codon. p1709a and p1709b sequence regions are underlined by straight lines and dashed lines, respectively.

The MntH is a major component of the Mn(II) uptake system. It is involved in oxidative stress resistance in many bacteria [10]. A *dr2539* null mutant of *D. radiodurans* showed increased intracellular manganese concentration and enhanced oxidative resistance, suggesting that DR2539 may be involved in the regulation of MntH [24]. To determine whether DR2539 binds to the *dr1709* promoter, we performed electrophoretic gel mobility shift assays (EMSA) in the presence of Mn(II). The *dr1709* promoter was amplified by PCR from the genome of *D. radiodurans* strain R1 (Fig. 1A). MntRs in *E. coli* and *S. enterica* were found to regulate the *mntH* by binding to the reverted repeat region of *mntH* promoters. In *D. radiodurans*, a similar reverted repeat region was found in the *dr1709* promoter. In order to confirm whether DR2539 binds to this region, we designed two pairs of primers: one pair was capable of generating *dr1709* promoter DNA fragments containing reverted repeat regions (dr1709b), and the other pair of primers was capable of generating *dr1709* promoter DNA fragments lacking the reverted repeat region (dr1709a) (Fig. 1A). As indicated by the gel shift, the DR2539 protein bound to the dr1709b promoter, and binding increased with protein concentration (Fig. 1B). In contrast, the DR2539 protein cannot bind to *dr1709* promoters that lack inverted repeat regions (Fig. S2A). This suggests that the binding between DR2539 and *dr1709* promoters depends on the presence of the inverted repeat region. The presence of EDTA in the EMSA reaction abolished binding to the *dr1709* promoter, suggesting that Mn(II) is necessary for DR2539–DNA binding (Fig. S2B). These results indicate that DR2539 specifically binds to the *mntH* promoter of *D. radiodurans*.

### Effects of Mn(II) and Fe(II) on DR2539 *in vitro*

Generally, the regulation of MntH genes involves iron-sensing by Fur regulatory proteins [18], [23]. In *D. radiodurans*, *dr0865* encodes the only Fur homologue. To investigate whether DR0865 might be involved in the regulation of the *mntH* gene, we expressed the DR0865 protein *in vitro* and performed DNA binding experiments. Unexpectedly, the EMSA results demonstrated that DR0865 cannot bind to the promoter of *dr1709* (p1709b is the full length version of *dr1709* promoter) (Fig. S2C). Current evidence supports the idea that MntR is a strictly Mn(II)-specific metalloregulatory protein, and that the repression of MntH by Fe(II) is always mediated by Fur [20], [21], [25]. We therefore wondered whether DR2539 can respond to both Mn(II) and Fe(II) *in vitro*. We studied the binding efficiency of DR2539 to the *dr1709b* promoter using EMSA in the presence of varying concentrations of Mn(II) and Fe(II). As shown in Fig. 1C, the binding activity of DR2539 increased as the concentration of Mn(II) rose from 25 µM to 150 µM. The same response was observed when Mn(II) was replaced by Fe(II) (Fig. 1D), indicating that the activity of DR2539 can be regulated by both Mn(II) and Fe(II) in a concentration-dependent manner.

### Effects of Mn(II) and Fe(II) on DR2539 *in vivo*

**Figure 2:**
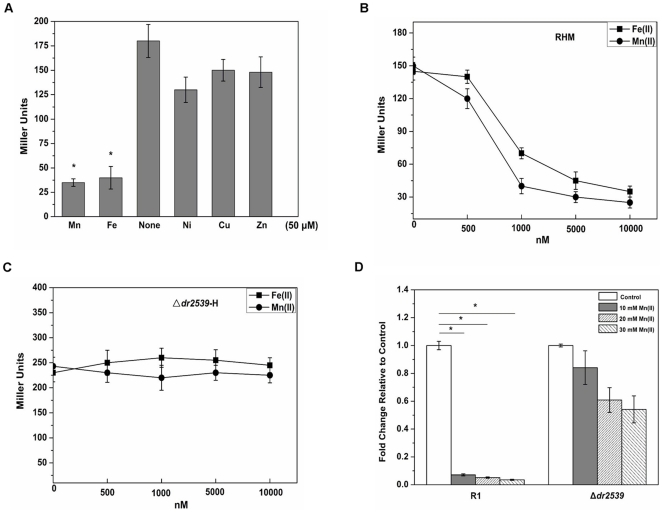
Mn(II) and Fe(II) modulate the binding activity of DR2539 *in vivo*. (A) Effects of divalent metals (50 µM) on expression of pRAZH in *D. radiodurans*. Data shown are the means ± standard deviations of three independent experiments. (B) Effects of Mn(II) (squares) and Fe(II) (circles) on the expression of pRAZH in wild-type samples expressing DR2539. (C) Effects of Mn(II) (squares) and Fe(II) (circles) on the expression of pRAZH in the *dr2539* null mutant. (D) Rea-time PCR analysis of the *dr1709* gene expression using *dr0089* as internal control gene. Longitudinal axes indicate the change fold of *dr1709* mRNA relative to controls. Control cells were cultured in medium without Mn(II). *, *P*<0.05 relative to control. The data are the means ± standard deviations of three independent experiments.

In order to confirm our results *in vitro*, the transcriptional regulation of the *mntH* promoter by different ions were tested by linking the *dr1709* promoter to the β-galactosidase gene *lacZ*. As shown in Fig. 2A, the activity of β-galactosidase was repressed dramatically when Mn(II) or Fe(II) was included in the growth medium, but not when Ni(II), Cu(II), or Zn(II) was added in the absence of Mn(II) and Fe(II). This strongly suggests that the expression of *D. radiodurans* MntH is regulated by both Mn(II) and Fe(II) *in vivo*. A *dr2539* null mutant was constructed to investigate whether the MntH repression induced by Fe(II) was actually mediated by DR2539. The expression of *lacZ* was repressed when wild-type bacteria were treated with different concentrations of Mn(II) or Fe(II) (Fig. 2B). On the other hand, Mn(II)- and Fe(II)-dependent repression of β-galactosidase was abolished in the *dr2539* null mutant, indicating that Mn(II)/Fe(II)-dependent repression of MntH (*dr1709*) was mediated by DR2539 (Fig. 2C). Using Real-time PCR analysis, we compared the expression levels of *dr1709* in the *dr2539* mutant and wild-type strains. As shown in Fig. 2D, the mRNA of the wild-type strain decreased dramatically when the concentration of Mn(II) increased relative to that cultured in media without additional Mn(II) (indicating control). No difference was observed in *dr2539* mutants, indicating that *dr2539* encodes the *dr1709* repressor.

### Binding of DR0865 to *mntABC* promoters

**Figure 3:**
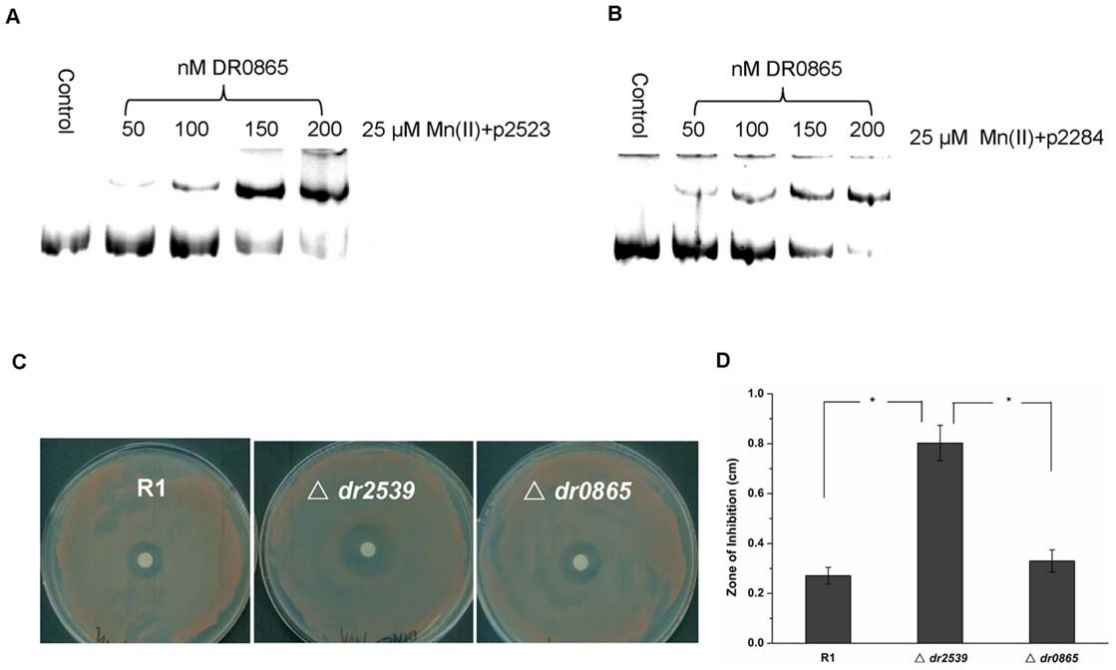
DR0865 binds to the promoter of MntABC in an ion-dependent manner. (A) and (B) DR0865 binding to p2523 and p2284 as the concentration of DR0865 increased. (C) Wild-type R1, *dr2539* null mutant (**Δ** *dr2539*), and *dr0865* null mutant (**Δ** *dr0865*) were cultured on TGY plates overlaid with filter discs saturated with 1 M solution MnCl_2_. (D) The zone of inhibition was measured from edge of disc after three days. *, *P*<0.05. Data represent the means±deviations of three independent experiments.

Because DR0865 cannot regulate MntH transcription, we turned our attention to *dr2523*, *dr2283*, and *dr2284*, which encode the other putative Mn(II) acquisition transporters. The EMSA results indicate that DR0865 binds to the promoters of *dr2523*, *dr2283*, and *dr2284* (*dr2283* and *dr2284* share one promoter), which encode the MntABC subunits in a manganese-dependant manner (Fig. 3A/3B). On the other hand, we found that DR2539 protein cannot bind to the promoters of *dr2283*, *dr2284*, or *dr2523* (data not shown). The manganese resistance assay showed that the *dr0865* null mutant is comparable to the wild strain while the manganese resistance of the *dr2539* null mutant decreased significantly (Fig. 3C/3D). These results imply that the MntABC in *D. radiodurans* may not be involved in manganese transport. Therefore, DR2539 may be the most important regulator of intracellular manganese homeostasis in *D. radiodurans*.

### Effects of H98Y mutation on the metal-sensing function of DR2539

**Figure 4:**
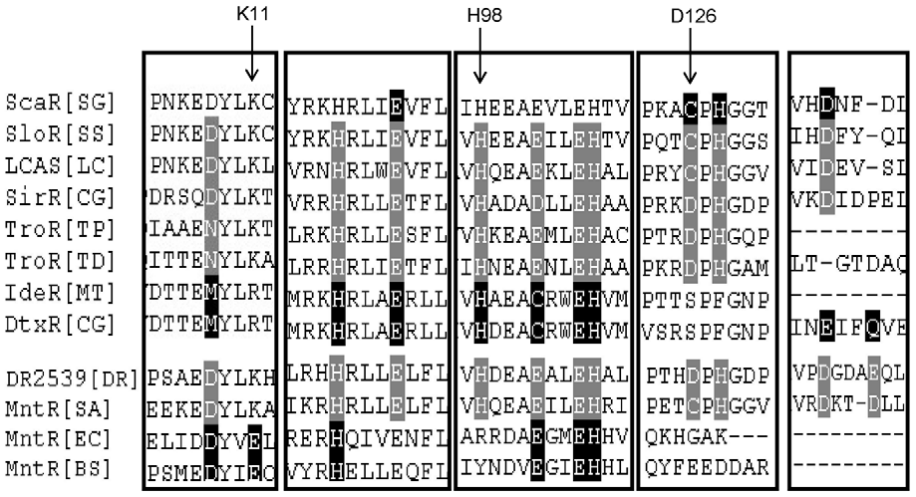
Sequence alignment of the metal binding sites of DR2539 with other DxtR/MntR family members. ScaR (*Streptococcus gordonii*), SloR (*Streptococcus suis*), LCAS (*Lactobacillus casei*), SirR (*Corynebacterium glutamicum*), TroR (*Treponema pallidum*), TroR (*Treponema denticola*), IdeR (*Mycobacterium tuberculosis*), DtxR (*Corynebacterium diptheriae*), DR2539 (*Deinococcus radiodurans*), MntR (*Staphylococcus aureus*), MntR (*Escherichia coli*), and MntR (*Bacillus subtilis*). The sequences were aligned using the CLUSTAL W software. Residues shaded with black represent metal-binding sites that have been studied while residues shaded with grey represent predicted metal binding sites.

**Figure 5:**
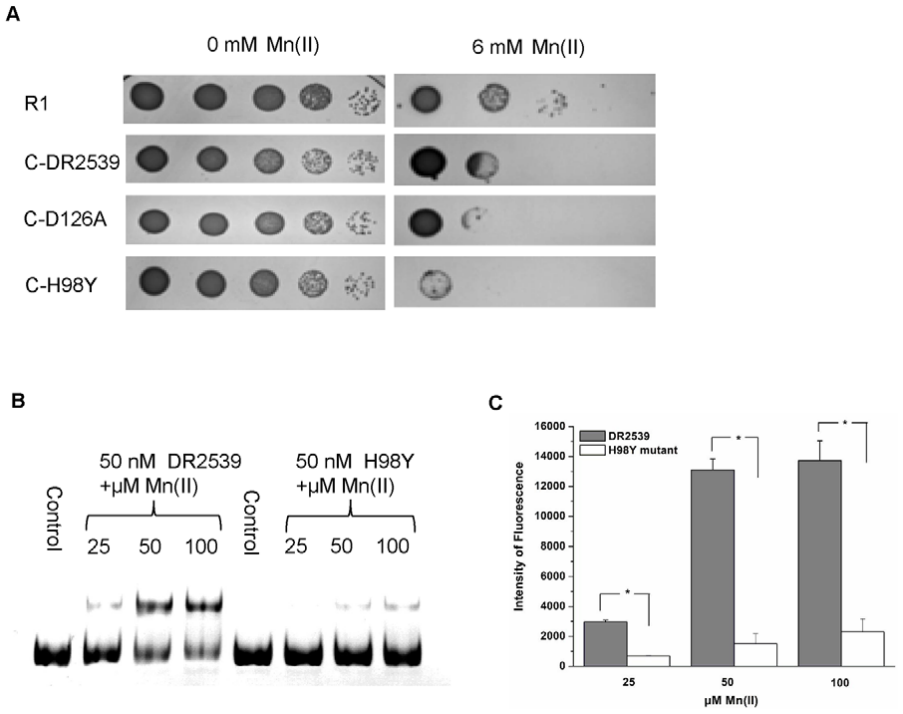
His98 plays an important role in DNA binding activity of DR2539. (A) 10 µl cell dilution was dripped on the TGY plate to which 6 mM of Mn(II) had been added. The cells were cultured for 3 days. (B) H98Y mutant and wild-type DR2539 proteins were incubated with p1709b at different concentrations of Mn(II). (C) Quantification of the fluorescence intensity of binding bands was performed using ImageJ. *, *P*<0.05.

The protein sequences of various members of the bacterial MntRs/DtxR family were aligned to define those structure features of DR2539 that could confer dual Mn(II)/Fe(II)-dependent functional regulation. Sequence alignment analysis indicated that DR2539 possesses a conserved His98 that is also present in Mn(II)/Fe(II)-dependent DtxR but absent from the MntH regulator (MntR). ScaR represents a new group of Mn(II)-responsive transcription factors, and its metal binding residues are conserved in DR2539 except Asp126 (Fig. 4). Site mutants H98Y and D126A were constructed to evaluate the metal binding mode of DR2539. These *dr2539* mutants were reverted to *dr2539* null mutants. The manganese resistance of the *dr2539* mutant complementation strain was examined in TGY plates supplemented with 6 mM manganese. As shown in Fig. 5A, the manganese resistance of the D126A complementation strain is comparable to that of the wild-type *dr2539* complementation strain. In contrast, the manganese resistance of H98Y complementation strain decreased dramatically.

To investigate the DNA binding activity of the H98Y mutant, the H98Y mutant protein was expressed *in vitro*, and the DNA binding activity was examined. The DNA binding activity of the H98Y mutant was found to be much weaker than that of wild type DR2539 at the same manganese concentration (Fig. 5B/5C). This shows that His98 may play a very important role in the function of DR2539 and that D126 may not be involved in the metal binding activity of DR2539.

## Discussion

*D. radiodurans* contains more intracellular Mn(II) than other bacteria [9]. It has been suggested that the high Mn/Fe ratio in *D. radiodurans* facilitates the pronounced resistance to extreme oxidative stress observed in this species [8], [13]. Intracellular Mn(II) is known to confer protection against oxidative stress and other forms of injury, including ionizing radiation [13]. We analyzed the genetic regulation of the MntH gene *dr1709* to study manganese homeostasis in *D. radiodurans*.

In *E. coli*, *mntH* transcription can be repressed either by the Mn(II)-dependent MntR or by the Fe(II)-dependent transcriptional regulator Fur [18]. The MntH of *S. enterica* has also been reported to be responsive to Fe(II) and the repression induced by Fe(II) was mainly mediated by Fur [23]. In contrast to previous studies, the gene *dr0865* was here found to encode the only Fur homologue in *D. radiodurans*, but it cannot bind to *dr1709* promoter. This indicates that Fe(II)-dependent regulation of MntH must be mediated by another Fe(II)-responsive transcriptional regulator. *D. radiodurans* DR2539 exhibited both Mn(II)-dependent and Fe(II)-dependent repression of *dr1709*. This explains the Fe(II)-dependence of MntH regulation in the absence of a functional Fur homologue.

The activation of metalloregulators is closely correlated with their structures. We compared the protein sequences of DR2539 to DtxR homologues and found substantial structural and functional differences. The Glu11 that provides two metal binding ligands in the MntR binuclear metal binding site is substituted by a basic amino acid (Lys) in DR2539. The presence of a positive charge at this position suggests that DR2539 cannot form a regular MntR-type metal binding site (Fig. 4). In addition, the His98 and SH3-like domains that are conserved in DtxR homologues (but missing in other MntRs) are present in DR2539.

In this paper, we observed that DR2539 responded to both Mn(II) and Fe(II), and the impact of H98Y and D126A on activity was assessed. The results indicate that the Mn(II) resistance of the C- H98Y complementation strain decreased significantly after exposure to Mn(II) and Fe(II), while the C- D126A strain was relatively resistant to Mn(II). A DNA binding assay implied that His98 is crucial to protein activity. This is different from previously reported MntR, because His98 is not involved in the metal binding of these proteins. This shows that DR2539 may represent a new subset of the DtxR family.

The disruption of the MntABC transporter could induce the accumulation of iron but could not change the manganese level in *D. radiodurans* [26]. Our results indicate that the DR0865 (Fur homologue) participates in the transcriptional regulation of the MntABC. The disruption of *dr0865* did not affect the manganese resistance of *D. radiodurans*. Therefore, we speculate that MntABC in *D. radiodurans* may be responsible for the intracellular iron efflux. However, there is still some controversy as to whether such an iron efflux mechanism exists in bacteria [27]. The function of DR0865 still needs to be elucidated.

Many studies have attempted to determine why MntR can selectively respond to Mn(II) while DtxR prefers to bind Fe(II). Here, we described a novel MntH repressor (DR2539) from *D. radiodurans*, and found it capable of responding to both Mn(II) and Fe(II). Moreover, the His98 in DR2539 is crucial to its activity. This is quite different from with results of previous reports on MntH repressors. However, crystallography analysis is still needed to confirm this and clarify the structure-function relationship.

## Materials and Methods

### Strains and media

All trains and plasmids used in this study are listed in Table S1. *D. radiodurans* strains were cultured at 30°C on TGY medium (0.5% Bacto tryptone, 0.1% glucose, 0.3% Bacto yeast extract) with aeration or on TGY plates supplemented with 1.2% Bacto agar. *E. coli* strain DH5α was used for propagation of plasmids and grown at 37°C on LB medium with appropriate antibiotics.

### Construction of pET-29b-*IF*-*dr2539* fusion expression vector

The *IF* and *dr2539* transcripts were cloned from *E. coli* using primers IFF/IFR and RFF/RFR (listed in Table S2). Then the PCR products were ligated separately into the pMD18-T simple vector (TaKaRa Biotechnology (Dalian) CO., Ltd, China) for sequencing (Invitrogen Corporation). The pMD18-T-*IF* plasmid was digested with *Nde*I and *Eco*RI and ligated to pET-29b digested with the same enzymes to form pETIF. The pMD18-T-*dr2539* vector was digested with *Eco*RI and *Hin*dIII and ligated to pETIF (digested with the same enzymes) to yield pET-29b-*IF*-*dr2539* (pIFMR), as shown in Fig. S1.

### Overexpression and purification of DR2539

Wild-type DR2539 was overexpressed in *E. coli* strain BL21 (DE3) (pLysS) transformed with pIFMR. Typically, 500 ml of LB medium was inoculated with 5 mL of a clone expressing DR2539 and cultured until OD_600_ was ≈0.6. Then 200 µM IPTG was added and the cultures were incubated for another 4 h. Cells were harvested by centrifugation and resuspended in 20 mL of lysis buffer (50 mM Tris-HCl (pH 8.0), 0.22 mg/ml lysozyme, 100 µM PMSF, and 10 mM DTT) and sonicated on ice. The lysate was centrifuged at 39,000×g for 30 min at 4°C. The clear supernatant containing the soluble protein was mixed with Ni-NTA slurry (QIAGEN) and rocked for 60 min at 4°C. The mixture was poured through a Ni-NTA column and washed with 50 ml of phosphate wash buffer (20 mM imidazole, 1 M NaCl, 10% glycerol, 50 mM Tris-HCl, pH 8.0). Purified His-IF-DR2539 protein was eluted with elution buffer (500 mM imidazole, 1 M NaCl, 10% glycerol, 50 mM Tris-HCl, pH 8.0). The imidazole and excess NaCl were removed by dialysis in 50 mM Tris-HCl, plus 300 mM NaCl and 10% glycerol. Then, the TEV protease, 5 mM EDTA and 0.1% DTT were added to the purified protein solution and digested overnight at 4°C. Imidazole (10 mM) was added to the digested protein suspension and the IF was removed by a Ni-NTA column. The protein was further purified with ion exchange chromatography and gel filtration chromatography. The DR0865 was purified using the same method. The purity of the proteins was analyzed with the SDS-PAGE (12% acrylamide) (Fig. S1).

### Electrophoretic mobility shift assay

Electrophoretic mobility shift assays (EMSAs) were performed as previously described with some modifications [28]. Briefly, FITC-labeled promoters were amplified from the genome of *D. radiodurans* strain R1 with primer pairs MHF/MHRa, MHF/MHRb, and MAF/MAR (listed in Table S2). The (NH_4_)_2_Fe(SO_4_) solution used for the DNA binding assay was prepared immediately before use to limit oxidation. The binding buffer contained 30 mM Tris-HCl (pH 6.8), 50 mM NaCl, and 5% glycerol. The DR2539 protein was incubated for 30 min at 4°C in EMSA binding buffer supplemented with 50 µg/mL calf thymus DNA, 50 µg/mL bovine serum albumin, Mn^2+^ or Fe^2+^ (at various concentrations), and 10 nM FITC-labeled promoter DNA fragments containing the promoter. After incubation, the reaction mixtures were analyzed on 5% nondenaturing polyacrylamide gels. The labeled DNA was detected using a LAS-3000 cooled CCD camera system (Fujifilm).

### Construction of promoter-lacZ transcriptional fusions and β-galactosidase assays

*D. radiodurans dr1709* gene promoter was amplified by PCR using the primer pair HPF/HPR (Table S2). The PCR products were ligated to pMD18-T simple vector for sequencing (Sango Biotech (Shanghai) Co., Ltd). Then, the *dr1709* gene promoter was digested with *Bgl*II and *Spe*I and further ligated into pRADZ vector yielding pRAZH [29]. The pRAZH vector was transformed into the *dr2539* null mutant as described previously [4], [24].

For β-galactosidase activity assays, cells were grown to OD_600_≈1.0 and permeabilized with Triton X100 as described previously [30]. β-galactosidase activity was assayed using standard methods. Activity was expressed in Miller units, defined as 1,000 times the scattering-corrected OD_420_ per OD_600_ of cells per minute.

### Real-time quantitative PCR

Real-time quantitative PCR was used to determine whether DR2539 modulates the transcription of *dr1709*. In short, cells were grown to OD_600_≈0.2 and then 10 mM, 20 mM, or 30 mM MnCl_2_ was added. Cells were harvested by centrifugation at 4000 rpm at 4°C after the OD_600_ was 0.4–0.45. Total RNA was extracted from 200 ml of cell cultures using TRIZOL Reagent (Invitrogen Corp., Carlsbad, CA, U.S.) after liquid nitrogen grinding and then treated with 10 units of RNase free DNase I (Promega, Mannheim, Germany). First-strand cDNA synthesis was carried out in 20 µl reaction mixtures containing 1 µg of each DNase I-treated and purified total RNA sample, combined with 3 µg of random hexamers. The Real-time PCR amplification used SYBR *Premix Ex Taq*™ (TaKaRa Biotechnology (Dalian) Co, Ltd, China) following the manufacturer's instructions. All assays were performed using the STRATAGENE Mx3005*P*™ Real-time detection.

### Site-directed mutagenesis of *dr2539* and complementation assays of the *dr2539* null mutant

Site-directed mutagenesis of *dr2539* was performed as described previously [31]. Briefly, *dr2539* was amplified by PCR using the primer pair MRF/MRR (Table S2) and cloned into pMD-18 simple vector. It was amplified using M126F/M126R (Table S2) as a primer and pMD-18-*dr2539* as a template. Amplified vector was treated with the enzyme *Dpn*I, which cleaves only when its recognition site is methylated. Following digestion, the DNA fragments were cloned into the pMD-18 simple vector. The D126A site mutation was confirmed by DNA sequencing. The confirmed pMD18-D126A was digested with *Nde*I and *Bam*HI and cloned into pRADK [32]. It was treated with the same enzymes to yield the plasmid vector pD126A. Identical methods were used to construct pH98Y using primers M98F/M98R (Table S2).

Plasmid vectors (pRKR, pD126A, pH98Y) were transferred into the *dr2539* null mutant as described previously [4]. In order to confirm the identities of the complementation strains, the genome of the complementation strain was extracted and PCR was conducted using the genome as a template and primer pair PradF/PradR (Table S2). The PCR product was sent for commercial sequencing.

### Manganese-sensitive assay

Complementation strains were cultured in TGY until OD_600_≈1.0. Cells were collected and diluted with PBS. Cell suspensions (10 µL) were dripped on plates with different concentrations of Mn(II). (A plate without Mn(II) was used as a control.) Plates were cultured for 3 days at 30°C. The results were captured using a scanner. For plate counting, 10^4^ and 10^5^ cells were inoculated on plates containing different concentrations of Mn(II) (Plates without Mn(II) were used as controls). The number of clones was determined after 3 days at 30°C.

## Supporting Information

Figure S1 **Overexpression and purification of the DR2539, H98Y mutant and DR0865 in** ***E.coli*** **.** (A) Schematic of the construction of the *IF*-*dr2539* fusion expression vector. (B) DR2539 was expressed as a fusion protein with solubility partner IF, and purified following digestion by TEV protease (1%) and Ni-NTA chromatography. Samples were analyzed by SDS-PAGE (12% acrylamide). Lane 1, Fusion expressed DR2539. Lane 2, Fusion expressed DR2539 treated with TEV. Lane 3, Purified DR2539 protein. Lane 4, Purified IF protein which was expressed by pETIF. MW, molecular size markers. (C) and (D) H98Y mutant of DR2539 and DR0865 proteins analyzed by SDS-PAGE (12% acrylamide).
(TIF)

Figure S2 **DR2539 binds to** ***dr1709*** **promoter depending on the present of inverted repeat region and Mn(II).** (A) *dr1709* promoter (p1709a) incubated with DR2539 in the presence of 25 µM Mn(II). (B) p1709b was incubated with DR2539 in the presence of 100 nM protein and 1 mM EDTA. (C) *dr1709* promoter (p1709b) incubated with DR0865 in the presence of 25 µM Mn(II).
(TIF)

Table S1 **Strains and plasmids used in this study.**
(DOC)

Table S2 **Primers used in this study.**
(DOC)
