# Supplementary figures and images for: Regulation of MntH by a Dual Mn(II)- and Fe(II)-Dependent Transcriptional Repressor (DR2539) in Deinococcus radiodurans
Source: PLoS One. 2012 Apr 16;7(4):e35057. doi: 10.1371/journal.pone.0035057 (PMC3327659; doi:10.1371/journal.pone.0035057)

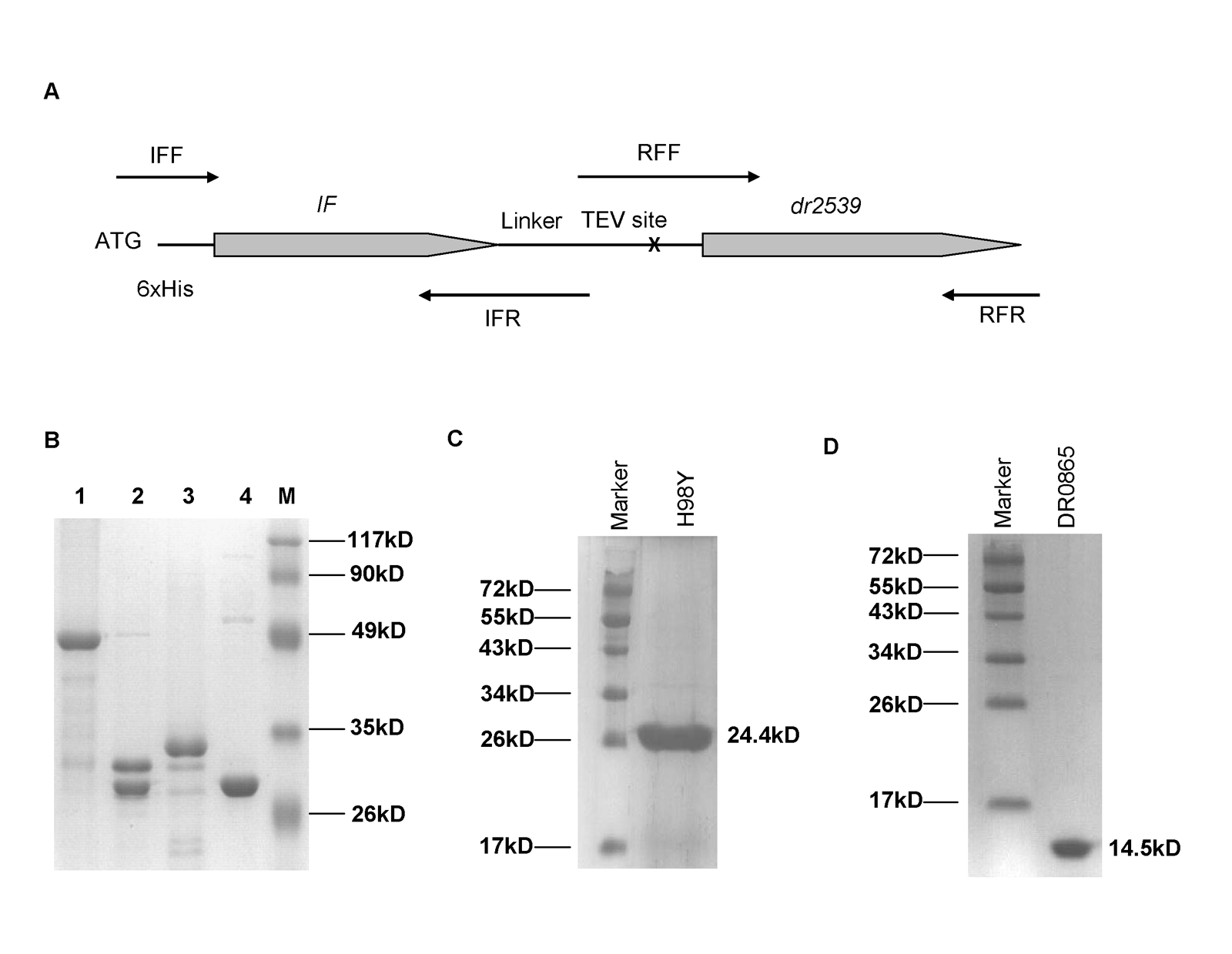

Supplement: Figure S1 — Overexpression and purification of the DR2539, H98Y mutant and DR0865 in E.coli . (A) Schematic of the construction of the IF-dr2539 fusion expression vector. (B) DR2539 was expressed as a fusion protein with solubility partner IF, and purified following digestion by TEV protease (1%) and Ni-NTA chromatography. Samples were analyzed by SDS-PAGE (12% acrylamide). Lane 1, Fusion expressed DR2539. Lane 2, Fusion expressed DR2539 treated with TEV. Lane 3, Purified DR2539 protein. Lane 4, Purified IF protein which was expressed by pETIF. MW, molecular size markers. (C) and (D) H98Y mutant of DR2539 and DR0865 proteins analyzed by SDS-PAGE (12% acrylamide). (TIF) [file pone.0035057.s001.tif]

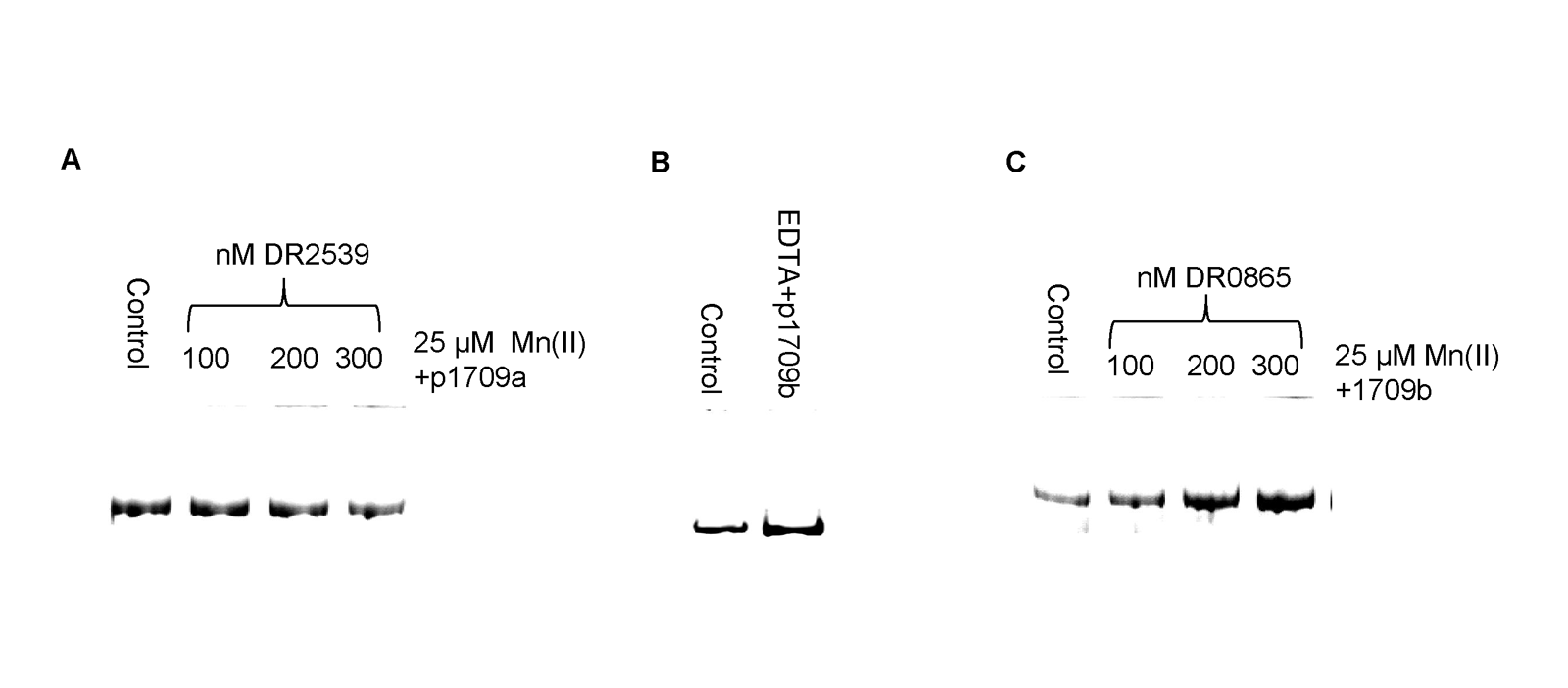

Supplement: Figure S2 — DR2539 binds to dr1709 promoter depending on the present of inverted repeat region and Mn(II). (A) dr1709 promoter (p1709a) incubated with DR2539 in the presence of 25 µM Mn(II). (B) p1709b was incubated with DR2539 in the presence of 100 nM protein and 1 mM EDTA. (C) dr1709 promoter (p1709b) incubated with DR0865 in the presence of 25 µM Mn(II). (TIF) [file pone.0035057.s002.tif]
