# Supplementary material for: Regulation of MntH by a Dual Mn(II)- and Fe(II)-Dependent Transcriptional Repressor (DR2539) in Deinococcus radiodurans
Source: PLoS One. 2012 Apr 16;7(4):e35057. doi: 10.1371/journal.pone.0035057 (PMC3327659; doi:10.1371/journal.pone.0035057)
Supplement: Table S1 — Strains and plasmids used in this study. (DOC) [file pone.0035057.s003.doc]

| **Strain or plasmid** | **Relevant marker** | **Reference or source** |
| --- | --- | --- |
| **Strains** | | |
| *E. coli* DH5α | Propagation for plasmid | Invitrogen |
| *E. coli* BL21(pLysS) | DR2539 expression strain | Invitrogen |
| *D. radiodurans* R1 | ATCC13939 | This lab |
| Δ*dr2539* | As R1, but *dr2539*::*kan* |  |
| Δ*dr0865* | As R1, but *dr0865*::*kan* | This study |
| RMH | R1 transformed with *pRAZH* | This study |
| Δ*dr2539*-H | Δ*dr2539* transformed with *pRAZH* | This study |
| C-DR2539 | Δ*dr2539* complemented with *pRKR* | This study |
| C-D126A | Δ*dr2539* complemented with *pD126A* | This study |
| C-H98Y | Δ*dr2539* complemented with *pH98Y* | This study |
| **Plasmids** | | |
| pMD18-T | TA cloning vector | Takara |
| pET-29b | Expression vector | Takara |
| pETIF | pET-29b derivative expressing *IF* solubility partner | This study |
| pIFMR | pET-29b derivative recombinated expressing *IF* and DR2539 | This study |
| pRADZ | Vector carrying a lacZ gene without a promoter |  |
| pRAZH | pRADZ derivative carrying *dr1709b* promoter | This study |
| pRADK | *E. coli*-*D. radiodurans* shuttle vector |  |
| pRKR | pRADK derivative expressing DR2539 | This study |
| pD126A | pRADK derivative expressing DR2539 (D126A site mutated) | This study |
| pH98Y | pRADK derivative expressing DR2539 (H98Y site mutated) | This study |
