# Supplementary material for: Regulation of MntH by a Dual Mn(II)- and Fe(II)-Dependent Transcriptional Repressor (DR2539) in Deinococcus radiodurans
Source: PLoS One. 2012 Apr 16;7(4):e35057. doi: 10.1371/journal.pone.0035057 (PMC3327659; doi:10.1371/journal.pone.0035057)
Supplement: Table S2 — Primers used in this study. (DOC) [file pone.0035057.s004.doc]

| **Primer** | **Sequence (5′ → 3′)** |
| --- | --- |
| IFF | acgtcatatgcaccaccaccaccaccacacagatgtaacg |
| IFR | gagcgaattcacgaccttcgatcactttgtctttttc |
| RFF | TTGAATTCGAAAACCTGTATTTTCAGGGAATGACCCGCACCCTCTCCCC |
| RFR | ACAAAGCTTAGCGAGGGGTGAGGGTGTCT |
| HPF | ACTGGACTAGTCAGGCTGGGAGAACGGGAATC |
| HPR | CAAAGATCTGAGCCTCTAGCAAATATGTGACAGCAC |
| MHF | GAGCCTCTAGCAAATATGTGACAGCAC |
| MHRa | TGTCCAACGGCGTGGGCTT |
| MHRb | CAGGCTGGGAGAACGGGAATC |
| MAF | CACTCCCCACACACGCA |
| MAR | AGAGTACCAACATGAGC |
| MRF | GCGCCATATGACCCGCACCCTCTCCCCCTCCG |
| MRR | ATATGGATCC AGCGAGGGGTGAGGGTGTCT |
| M126F | ccccacccatgccccgcacggcg |
| M126R | cgccgtgcggggcatgggtgggg |
| M98F | gctcgacgaggtgtatgacgaggccgagg |
| M98R | CCTCGGCCTCGTCATACACCTCGTCGAGC |
| PradF | CAGCTTCGGTCAGTTGACATT |
| PradR | TTTGATTGGACTAAAGACATACCCT |
